# Spatiotemporal bio-shielding of bacteria through consolidated geometrical structuring

**DOI:** 10.1038/s41522-022-00302-2

**Published:** 2022-05-09

**Authors:** Satish Kumar Rajasekharan, Moshe Shemesh

**Affiliations:** Department of Food Science, Institute of Postharvest Technology and Food Sciences, Agricultural Research Organization (ARO), The Volcani Institute, Rishon LeZion, 7528809 Israel

**Keywords:** Applied microbiology, Biofilms, Microbial communities

## Abstract

The probiotic bacterium *Lactobacillus plantarum* is often reckoned as a ‘generalist’ for its ability to adapt and survive in diverse ecological niches. The genomic signatures of *L. plantarum* have shown its intricate evolutionary ancestry and dynamic lifestyles. Here, we report on a unique geometrical arrangement of the multicellular population of *L. plantarum* cells. Prominently, a phenomenon of the cone-shaped colony formation and V-shaped cell chaining are discovered in response to the acidic-pH environment. Moreover, subsequent cold stress response triggers an unusual cellular arrangement of consolidated bundles, which appeared to be independently governed by a small heat shock protein (HSP 1). We further report that the V-shaped *L. plantarum* chaining demonstrates potent antagonistic activity against *Candida albicans*, a pathogenic yeast, both in vitro and in a *Caenorhabditis elegans* co-infection model. Finally, we deduce that the multifaceted traits manifested by this probiotic bacterium is an outcome of its dynamic flexibility and cellular heterogeneity.

## Introduction

Versatile adaptability of *Lactobacilli* into diverse ecosystems defines the uniqueness of these species^1^. While these bacteria are frequently associated with dairy products, invertebrates, and craniate, they also form a significant part of human microbiota^2^. These species are known to stimulate immunomodulatory effects and evince belligerent traits against deleterious bacterial or fungal pathogens^3–5^. Specifically, a probiotic bacterium *Lactobacillus plantarum* displays diverse morphological phenotypes and a remarkable ability to acclimatize to external settings, qualify it for wide-ranging medicinal and industrial applications^5^.

Undoubtedly, *L. plantarum* is a prospective probiotic bacterium in the food and supplements industry^6^. The capability of multicellular behaviour by this bacterium appears to be an integral factor contributing to its applicability in the food matrices as well as within human host^7,8^. In addition, *L. plantarum* successfully colonizes intestinal mucosa and co-exists with other beneficial bacteria in the human gut^8^. Collectively, probiotics and the indigenous bacterial populations alter the gut microbiome’s overall structure that offers multifarious health benefits to the human host^5^. For instance, establishment of the commensal bacteria is often thought to either disassemble the preformed biofilms of gut pathogens^9^ or combat them by triggering host immune response^10^. Furthermore, probiotics may ameliorate several morbid conditions of metabolic disorders, such as diabetes and obesity, as well as several gut disorders. Some of the probiotics function as psychobiotics as well, yielding cognitive benefits^11^.

*L. plantarum* is one of the unique species of *Lactobacillus* genus known to exhibit multifaceted population heterogeneity^12^. The cells of *L. plantarum* manifest a strong ability to auto-aggregate depending on external environments and nutrient availability^13^. Auto-aggregation or co-aggregation is an exciting adaptation tactic that potentiates the probiotic cells to combat harmful pathogens. A probiotic *Lactobacillus gasseri* was shown to adhere to the human intestinal mucosa by auto-aggregation, creating a protective blanket, which prevents pathogen colonization. In addition, several strains of *L. plantarum* are routinely assessed for their auto-aggregation abilities citing its effectiveness for pathogen control^14^.

Tolerance to acidic conditions is yet another adaptation response manifested by *Lactobacilli* to curtail pathogen growth in a microbiome. In general, cells of *L. plantarum* are well-adapted to withstand diverse pH-induced stresses. They grow well at sub-optimal pH conditions (pH 5.0), which may trigger a pre-adaptation response eliciting the cells to swiftly adjust to the stressful environment in a better way^15^. Other *Lactobacilli* are reported to tolerate extremely low-pH stresses by arresting growth and favouring colonization. For instance, *L. acidophilus* cultured in pH 3 revealed enhanced ability to survive and adhere to human intestinal cells^16^. Other studies have shown that the low pH (3.5–4.5) triggers *L. plantarum* upsurge in the human vaginal microbiome^17^. The proliferation, in turn, aids *L. plantarum* to exhibit complete dominance during competition within the vaginal microbiota. Overall, phenotypes displayed by *L. plantarum* in response to acidic-pH may account for the remarkable adaptability to survive and combat pathogens thereof, which seems sturdily connected to its origins and distribution.

Recently, the genomic analysis revealed that the genus *Lactobacillus* relishes three different forms of lifestyles: free-living, nomadic, and host-adapted^2,5^. The nomadic lifestyle, for instance, enables *Lactobacilli* to grow in diverse environments^2^. Thus, understanding the nomadic ancestry of *L. plantarum* might provide a clearer insight into cellular heterogeneity and dynamic adaptability. This understanding can be realized by examining the animal-derived probiotic model, as it tends to manifest seemingly unique traits like enhanced digestive enzymes and superior antagonistic activities^18^. Their subtypes are likely dispersed and drifted into different ecosystems (via animal excrement), through paving a way for further evolution and adaptation.

In this study, we characterize morphological traits of the multicellular population of *L. plantarum* through a unique geometrical arrangement facilitating its survivability under different stress conditions. Furthermore, we provide mechanistic evidence of how the acidic-pH adaptation of this bacterium is tightly linked to its vast antagonistic potential against pathogenic microbial species, for intense *Candida albicans*, in an in vivo *Caenorhabditis elegans* model.

## Results

### Spatiotemporal establishment of conic colonies is governed by an adaptation response of *L. plantarum* cells to acidic-pH

**Fig. 1:**
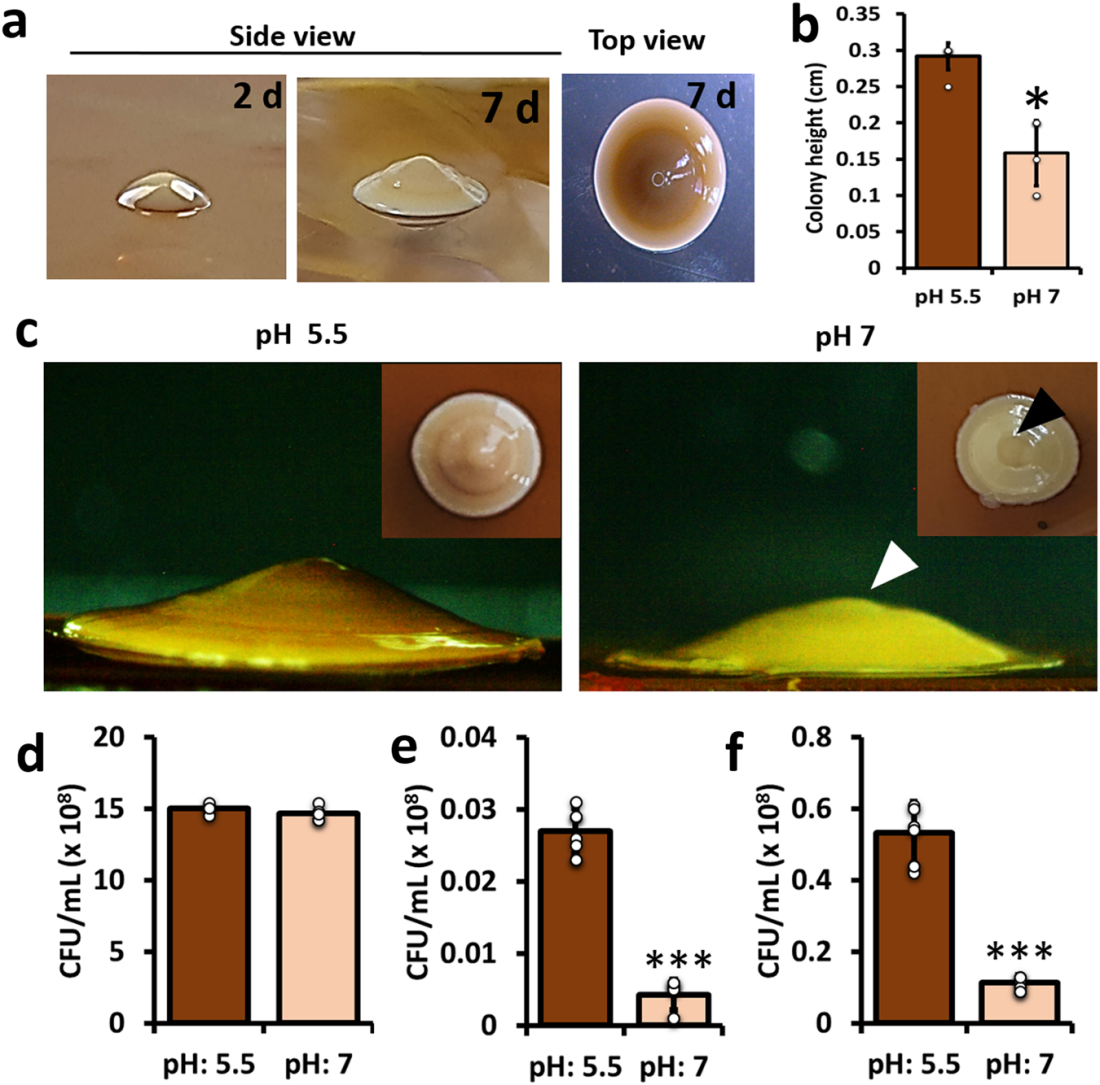
Spatiotemporal establishment of conic-shaped colonies in *L. plantarum*. **a** Colonies of *L. plantarum* 3297 were generated onto the MRS hard agar plates (pH 5.5) following 2 and 7d incubation at 37 °C. Scale bar: 0.2 cm. **b** Estimation of the *L. plantarum* 3297 colony heights generated on the MRS hard agar plates with different pH conditions. The graph shows the means ± SEMs of 6 individual data points, obtained from two independent measurements. *P < 0.05 vs. the control. Here, the colonies grown at pH 5.5 are taken as control compared to those grown at pH 7. **c** Effect of ΔpH on conic tip formation and colouration of the *L. plantarum* colonies. White and black arrowhead show the abolished tip. Scale bar: 0.1 cm. **d** The CFU quantitation of *L. plantarum* 3297 cells grown at pH 5.5 and 7 after 7 d of incubation on MRS hard agar. The graph shows the means ± SEMs of individual data points, obtained from two independent measurements. *P > 0.05 vs. the control. Here, the colonies grown at pH 5.5 are taken as control compared to those grown at pH 7. **e** The CFU quantitation of *L. plantarum* 3297 cells exposed to desiccation stress. The cells were prior grown on MRS hard agar at pH 5.5 or 7 for 7d. After incubation, the conic colonies were directly transferred to the desiccation unit (at 40% relative humidity and 30 °C for 4 d). The graph shows the means ± SEMs of 9 individual data points, obtained from three independent measurements. ***P < 0.001 vs. the non-treated controls. **f** The CFU quantitation of *L. plantarum* 3297 cells exposed to desiccation stress. The cells were prior grown on MRS hard agar at pH 5.5 or 7 for 7d. After incubation, the conic colonies were directly transferred to the desiccation unit (at 40% relative humidity and 30 °C for 20 h). The graph shows the means ± SEMs of 9 individual data points, obtained from three independent measurements. ***P < 0.001 vs. the non-treated controls.

This investigation was initiated following the observation of unusual cone-shaped colonies formed by *L. plantarum* on the MRS air–agar interface, which were triggered by acidic pH (Fig. 1). The highly structured small colonies expanded radially and aerially, reaching finite size with a circumferential diameter of 0.416 ± 0.02 cm (Supplementary Fig. S1a); growth ceased despite the availability of nutrients in the vicinity. The most straightforward interpretation of the observed phenomenon was that the *L. plantarum* cells found a way for aerial expansion within the colony during the growth in acidic pH. On average, a 7d old colony measured an aerial height of 0.3 cm ± 0.16 cm. We also noted the brown-coloured deposits that were apparent on the outer surfaces of these colonies (Fig. 1a and supplementary Fig. S1b). The intensity of the brown coloration increased with incubation time (Supplementary Fig. S2a), which indicated a possible correlation with cellular survivability (Supplementary Fig. S2b). The numbers of dead cells were significantly higher in 15d old colonies than observed in 5 or 7d colonies (Supplementary Fig. S2c). The mechanism of cell death in colonies could be attributed to ROS accumulation and instantaneous cell death (Supplementary Fig. S3). Furthermore, the colonies formed at pH 7 were devoid of brown deposits, resembling a frustum without a conical tip and the reduced colony height (Fig. 1b, c). Despite the phenotypic heterogeneities during growth in altered pH, the viable cell counts remained comparable within the tested colonies (Fig. 1d). These observations indicated that the brown deposits and conic tip formed at pH 5.5 are colony structures developed by the *L. plantarum* cells during adaptation to acidic-pH.

### The cone-shaped colonies accentuate a multi-stress response in cells within

Having linked the cone-shaped structure and brown deposits to the acidic-pH adaptation, we next sought to understand its biological significance. We hypothesized that the observed highly structured spatiotemporal colonization would provide a multi-stress response for the bacterial population. To explore this notion, we exposed the colonies to secondary desiccation stress and assessed the survivability of the cells inside the colonies. The desiccation tolerance was higher in colonies grown at acidic-pH (pH 5.5) than at elevated pH (pH 7) (Fig. 1e, f), signifying that the conic-shaped structure imparts some bio-shielding machinery. The possible interpretation of the observations is that brown-coloured deposits and conic-shaped geometry of the colonies (grown at pH 5.5) might help the cells to improve survivability during subsequent stress.

### The cold-shock response triggers consolidated bundle formation

**Fig. 2:**
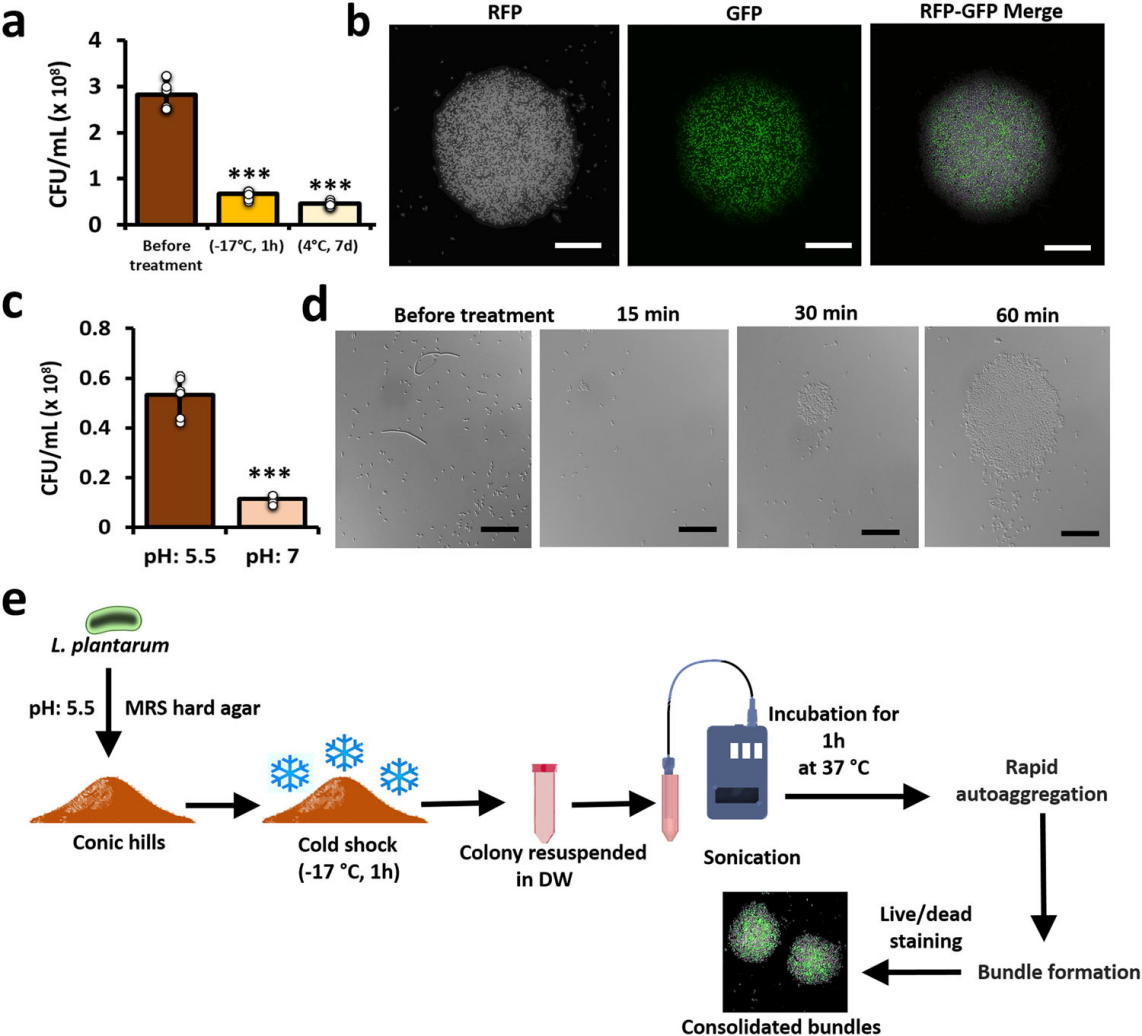
Influence of cold stress on survivability of *L. plantarum* grown in conic colonies. **a** The CFU quantitation of *L. plantarum* 3297 conic colonies following exposure to cold stress. The cells were grown on MRS hard agar (pH 5.5) for 7d and transferred to cold stress conditions (−17 °C) for 1 h. After incubation, the colonies were resuspended in phosphate-buffered saline (PBS), vortexed, diluted, and plated on fresh MRS agar plates. Similarly, the colonies were also incubated for 1 week at 4 °C and assessed subsequently. The graph shows the means ± SEMs of 9 individual data points, obtained from three independent measurements. ***P < 0.001 vs. the non-treated controls. **b** Dual staining (SYTO™ and propidium iodide (PI)) of consolidated bundles that was induced following frozen stress (−17 °C, 1 h). Scale bar: 20 µm. The cells stained with PI (true-colour: red) are false-coloured with grey. **c** The CFU quantitation of *L. plantarum* 3297 conic colonies following exposure to frozen stress. The cells were grown on MRS hard agar pH 5.5 or 7 for 7d and transferred to cold conditions (−17 °C) for 1 h. After incubation, the colonies were resuspended in phosphate-buffered saline (PBS), vortexed, diluted, and plated on fresh MRS agar plates. The graph shows the means ± SEMs of 9 individual data points, obtained from three independent measurements. ***P < 0.001 vs. the non-treated controls. **d** Time-course analysis of cellular aggregation of *L. plantarum* 3297 cells from conic colonies to form consolidated bundles (within 1 h). Scale bar: 20 µm. **e** Schematic experimental setup that led to the sighting of consolidated bundles. The cells stained with PI (true-colour: red) are false-coloured with grey.

Since cold-shock is regarded as one of the substantial stresses for the bacterial cells, we next tested whether the cone-shaped colonies would provide increased survivability during exposure to the cold stress. The cellular cryotolerance drastically fell in the cold-stressed (−17 °C for 1 h) colonies (Fig. 2a), nonetheless the survivability of cells grown at the acidic-pH stressed colonies was comparatively higher (Fig. 2c). In the course of performing the cold stress experiments, several exciting observations were revealed. The most intriguing finding was the sighting of unique consolidated circular bundles in acidic-pH (pH 5.5) stressed colonies following a freeze-thaw challenge (Fig. 2b). Time-lapse imaging revealed that cells harvested from the conic colony rapidly auto-aggregated to form the circular bundles (Fig. 2d, e). Notably, the bundles consisted of live and dead cells, with the former localized at the centre and the latter occupying the periphery. This observation indicates a possible cellular cryoprotective phenomenon, which apparently needs further investigation.

### A heat shock protein 1 (HSP1) is a keystone factor for consolidated bundling during cold stress

**Fig. 3:**
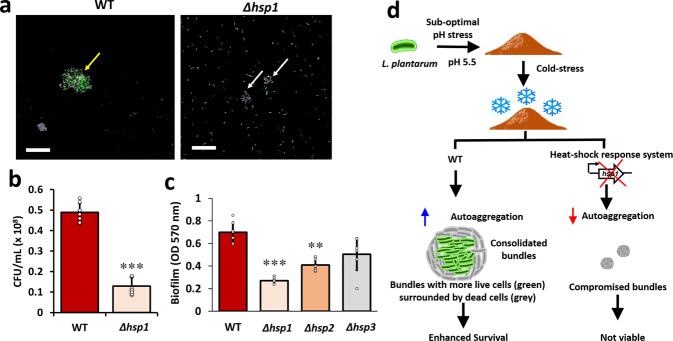
The heat-shock proteins are involved in cold shock stress response. **a** Consolidated bundles of wild-type *L. plantarum* WCFS1 or *hsp1* mutant (*Δhsp1*). Scale bar: 20 µm. The cells stained with PI (true-colour: red) are false-coloured with grey. Yellow arrow indicate consolidated bundles and white arrows indicate compromised bundles. **b** Colony-forming units (CFUs) of wild-type *L. plantarum* WCFS1 or *hsp1* mutant following exposure to frozen stress. The cells were grown on MRS hard agar pH 5.5 for 7d and transferred to cold stress conditions (−17 °C) for 1 h. After incubation, the colonies were resuspended in phosphate-buffered saline (PBS), vortexed, diluted, and plated on fresh MRS agar plates. The graph shows the means ± SEMs of 9 individual data points, obtained from three independent measurements. ***P < 0.001 vs. the non-treated controls. **c** Crystal violet quantification of biofilms formed by *L. plantarum* WCFS1, and *hsp* mutants (*Δhsp1, Δhsp2*, or Δ*hsp3*) on polystyrene surface. The cells were incubated at 37 °C for 24 h without shaking, following which the cells were stained with 0.4% crystal violet and read at OD_595_. The graph shows the means ± SEMs 6 individual data points, obtained from two independent measurements. ***P < 0.001 vs. the non-treated controls. Here cells grown at pH 5.5 are taken as a control. **d** Mechanistic flowchart depicting the involvement of heat-shock protein response in governing the multifaceted traits manifested by *L. plantarum* in response to acidic-pH.

Small heat shock proteins (sHSP) have an influential role during heat or cold acclimations^19^. Probiotic *Lactobacilli* have typically have either one or two sHSP genes, with the exemption of *L. plantarum* that harbors three sHSP genes, namely *hsp1, hsp2*, and *hsp3*. Lately, the involvement of *hsp1* in cryoprotecion was stated^19^. We, therefore, explored a possible link between sHSP and consolidated bundle formations by *L. plantarum*. Using *hsp1* knockout mutant (*Δhsp1*), we show that the bundles’ size was relatively reduced (Fig. 3a and Supplementary Fig. S4). Besides, *Δhsp1* mutant also displayed reduced survivability during the freeze-thaw challenge (Fig. 3b), and formed poor biofilms on polystyrene surfaces (Fig. 3c). In contrast, *Δhsp2* or *Δhsp3* mutants formed either moderate or substantial biofilms, respectively, and did not show a significant difference in the freeze-thaw challenge. Hence, the findings up to now suggest the involvement of HSP1 in governing the consolidated bundle formation and cellular protection during the cold stress (Fig. 3d).

### Formation of the consolidated circular bundles is triggered by signalling molecules derived from the cold-shock colony filtrate (CSCF)

**Fig. 4:**
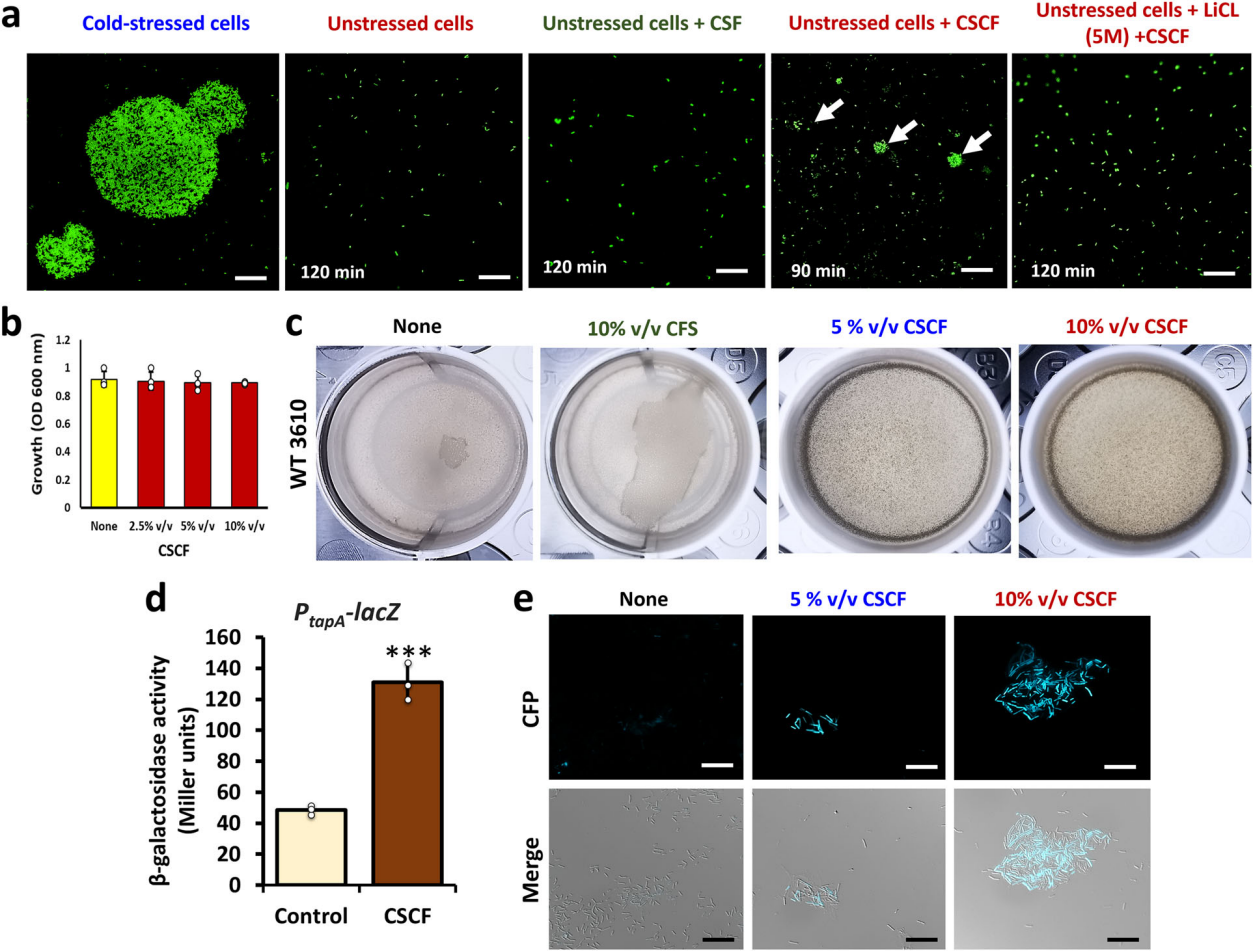
Effect of CSCF from *L. plantarum* conic colonies on multicellular behaviour by probiotic *Bacilli*. **a** Effect of cold-shock colony filtrates (CSCF) on unstressed *L. plantarum* 3297 cells from conic colonies. Lithium chloride (5 M) is chemical that prevent cellular aggregation. Scale bar: 20 µm. White arrows indicate small bundles. **b** Effect of CSCF (10% v/v) on the growth of *B. subtilis* NCIB 3610 incubated at 37 °C for 24 h at 150 rpm. The graph shows the means ± SEMs of three measurements. *P > 0.05 vs. the control. **c** Effect of CSCF (10 % v/v) on pellicle formation of *B. subtilis* NCIB 3610 incubated at 30 °C for 48 h without shaking. **d** The β-galactosidase activity assay (measured in Miller units) in *B. subtilis* (YC121) cells harboring *P*_*tapA*_*-lacZ* transcriptional reporter. The graph shows the means ± SEMs of three measurements. **P < 0.01, ***P < 0.001 vs. the non-treated controls. **e** Effect of CSCF (10% v/v) on bundle formation in *B. subtilis* (YC189), harboring a transcriptional reporter (*P*_*tapA*_*-cfp*) indicating on the matrix gene expressionf. Scale bar: 20 µm.

We further hypothesized that the auto-aggregation of the cold-stressed colony cells into the consolidated circular bundles could be triggered by some unidentified, but self-produced signalling molecules. We tested whether the cold-shock colony filtrate (CSCF) could trigger circular bundle formation in unstressed colony cells to address this concept. We noticed a formation of small-to-medium-sized circular bundles within 90 or 120 min (Fig. 4a), confirming our hypothesis. The phenomenon was prevented in cells pre-incubated with lithium chloride (5 M) (Fig. 4a), which removes the S-layer proteins of the bacteria, and renders the cells inefficient to receive any external signals^20^. The circular bundles were absent also when the colony filtrates (CF) from unstressed colonies (Fig. 4a) or in CSCF exposed to heat treatment (60 °C for 30 min) (Supplementary Fig. S5). Overall, these results reveal that the cold stress potentiates *L. plantarum* cells to release pre-accumulated signals that coordinate cellular aggregation.

### CSCF activates a heterologous multicellular aggregation in *Bacillus subtilis*

Lately, *B. subtilis* was shown to be involved in a symbiotic relationship with *L. plantarum*^21^. We hypothesized that the CSCF produced by *L. plantarum* cells would induce multicellular behaviour in *B. subtilis* through establishing a cross-talk between these probiotic species. We thus examined supplementation of the CSCF at various doses (1–10% volume per volume (v/v)) on *B. subtilis* response during growth in Lysogeny broth (LB) medium. A notable induction in pellicle formation by *B. subtilis* at specific concentrations (5 or 10% v/v) was observed (Fig. 4c), without affecting the bacterial growth (Fig. 4b and Supplementary Fig. S6). The *β*-galactosidase and fluorescent microscopic assays measuring the *tapA* (the major operon, required for polymerization of TasA amyloid fibers and their proper anchoring on cell surface toward biofilm formation) expression confirmed the activation of matrix production in response to the CSCF derived from *L. plantarum* cells (Fig. 4d, e). In addition, CSCF does not possess any suicidal effect on *L. plantarum* itself, and CF did not induce pellicle formation (Fig. 4c). The most conceivable explanation is that the putative signals are accumulated within the conic colonies and are released following cold stress disruption. The phenomenon could explain the triggering effect of the CSCF toward cellular aggregation (Fig. 4a, e).

### Low pH triggers the formation of unique V-shaped geometric cell chains

**Fig. 5:**
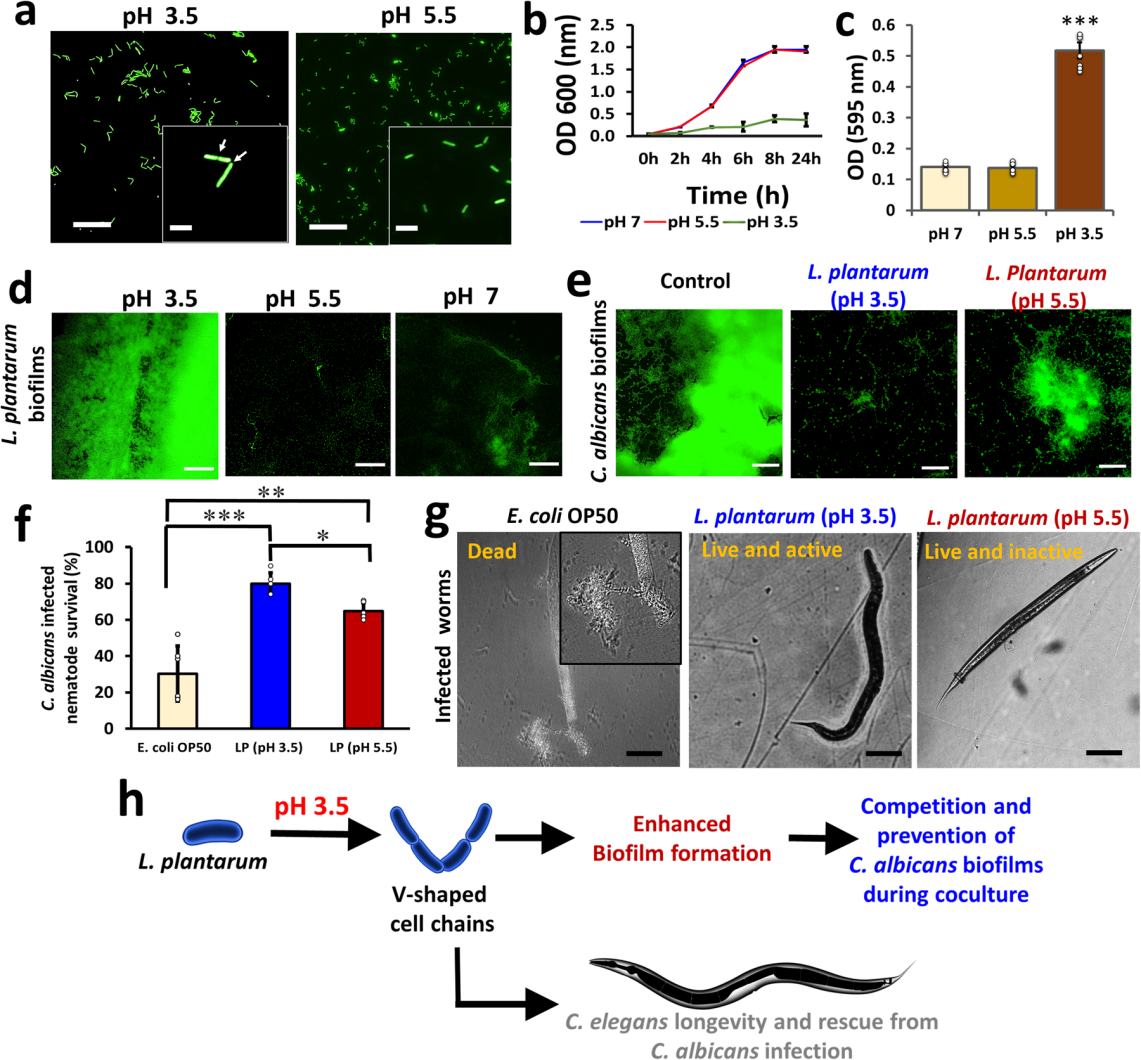
Effect of acidic pH on the *L. plantarum* geometric structuring and subsequent biofilm formation accompanied with antagonistic activity against *C. albicans*. **a** Phenotypic appearance of *L. plantarum* 3297 cells grown at pH 3.5 or 5.5 in liquid MRS. Scale bar: 20 µm. White arrows indicate septation, inset scale bar: 2 µm. **b** Growth curves of *L. plantarum* grown at either pH 3.5 or 5.5. The cells were incubated at 37 °C for 24 h. **c** Crystal violet quantification of *L. plantarum* biofilms on polystyrene surface. The cells were incubated at 37 °C for 24 h without shaking, following which the cells were stained with 0.4% crystal violet and detected at OD_595_. The graph shows the means ± SEMs of 9 individual data points, obtained from three independent measurements. ***P < 0.001 vs. the non-treated controls. Here cells grown at pH 5.5 are taken as a control. **d** Fluorescent microscopic images of *L. plantarum* biofilms on polystyrene surface stained with SYTO™ 9. Scale bar: 200 µm. **e** Effect of live probiotic cells (*L. plantarum* cells grown at either pH 3.5 or 5.5) on mature *C. albicans* biofilms in in vitro conditions. *C. albicans* biofilms were grown on polystyrene plates. After 8 h of incubation, the broth containing the planktonic cells were removed, replaced with PBS (controls) or live *L. plantarum* in PBS, and incubated again for 24 h. Biofilm formation was then assessed microscopically after staining with SYTO™ 9. Scale bar: 200 µm. **f** Survival rates of nematodes (that was prior fed with *L. plantarum* cells grown at either pH 3.5 or 5.5 or *E. coli* OP50) infected with *C. albicans*. The graph shows the means ± SEMs of 9 individual data points, obtained from three independent measurements. **P < 0.01, and ***P < 0.001 vs. the *E. coli* OP50 controls, ***P < 0.05 vs. *L. plantarum* at pH 5.5. **g** The microscopic image of adult *C. elegans* exposed to *C. albicans* and subsequently recused with *E. coli* OP50 (control) or *L. plantarum* (pH 3.5 or 5.5) feed. Inset shows *C. albicans* hyphae that ruptured and killed the nematode in *E. coli* OP50 fed group. Scale bar: 200 µm. **h** A scheme depicting the formation of V-shaped cell chains and their pertinence.

We further investigated the adaptive morphology of *L. plantarum* cells during the transition to low pHs. Intriguingly, cells grown at particularly acidic-pH (pH 3.5) displayed unique V-shaped cellular structures (Fig. 5a), though they showed slower growth rates compared to the cells grown at elevated pHs (Fig. 5b). The finding led us to assume that these cells retort to low-pH stress by stalling cell division by locking them in a V-shaped conformation consisting of four undivided cells. Besides, we found a “bottle effect” when the low pH grown cells retained their V-shaped structures while forming robust biofilms on polystyrene and glass surfaces (Fig. 5c, d & Supplementary Fig. S7). Collectively, these results demonstrate a survival mode of growth with increased resistance, which could be linked to biofilm formation.

### The V-shaped *L. plantarum* cells restrain *C. albicans* virulence and enhance *C. elegans* longevity

It has been reported that low pH (3.5–4.5) favours *Lactobacilli* dominance over some pathogens^17^. We thus tested if the V-shaped cells could potentially mitigate a medically important yeast pathogen, *C. albicans*. We tested the effect of V-shaped *L. plantarum* cells on the *C. albicans* biofilms in in vitro and *C. elegans* model. *L. plantarum* cells (grown in media with adjusted pH 3.5, or 5.5), was added (in potato dextrose broth (PDB)) to an 8 h biofilms of *C. albicans* and monitored its ability to stall biofilm maturation. We found that the live cells effectually mitigate the biofilms of *C. albicans* (Fig. 5e). Next, adult *C. elegans* were fed with *L. plantraum* (grown in media with either pH 3.5 or 5.5) and then infected with *C. albicans*. In control (*E. coli* OP50 fed) groups, *C. albicans* intensified nematode mortality rates (Fig. 5f) and effectively killed the nematode by piercing the cuticle (Fig. 5g, inset). In contrast, nematodes fed with *L. plantarum* cells exhibited better survival rates (Fig. 5f and g), particularly the groups fed with V-shaped *L. plantarum* grown in medium with adjusted pH 3.5 (Fig. 5f). Uninfected nematode survival rates are shown in Supplementary Fig. S8. The best interpretation for the observed phenotypes is that the *L. plantarum* cells likely colonize *C. elegans* intestine, which mitigate the *C. albicans* virulence. It also ties to our previous results that at pH 3.5 *L. plantarum* forms vigorous biofilms, via exhibiting unique V-shaped conformations (Fig. 5a, Supplementary Fig. S7), and apparently mitigate the colonization of *C. albicans* within *C. elegans*, through enhancing *C. elegans* longevity.

## Discussion

The key finding of this study is related to the unusual cone-shaped colonies (Fig. 1a, c) formed by the *L. plantarum* dairy isolate that appear to be associated with acidic-pH adaptation response. Though a vertical colony growth on hard agars is relatively unusual in *Lactobacilli* genera, it has been recently investigated in a non-motile and non-EPS producing *Escherichia coli* using a 3D agent-based model^22^. The group used computational simulations to show that the mechanical forces, and not the nutrients, induce these aerial structures. Other studies with *Pseudomonas fluorescens* Pf0-1 have demonstrated that the bacteria use secretions to push and expand vertically^23^. *Lactobacilli* is a taxonomically complex genus with some species displaying dynamic adaptability, survivability, and nomadic lifestyles^2^. Owing to these flexibilities, it is not surprising to witness aerial growth in *L. plantarum* colonies.

We next questioned the robustness of the cone-shaped colonies by challenging them to multiple stresses like the cold or desiccation stresses. Probiotics often need to endure stressful challenges during processing in food or pharmaceutical industries^24^. These practices are detrimental as a significant portion of the cells lose viability. Cold stress, for instance, is characterized by combination of multiple stresses that constantly challenge the bacteria, including the formation of ice crystals, osmotic imbalance, and dehydration^25^. *L. plantarum* has been known to tolerate cold-stress^24^. Exposing the conic *L. plantarum* colonies to extreme cold stress revealed unique consolidated circular bundles (Fig. 2b). Auto-aggregation and co-aggregation of probiotic cells are often regarded as beneficial traits^13^. The former halts adherence of pathogens to the intestinal mucosa, while the latter facilitates pathogen clearance or attenuation. The formation of consolidated bundles might resemble auto-aggregation, through activating rapid and self-reliant machinery to conserve the surviving bacterial population. That said, the mechanism governing auto-aggregation in probiotic LABs is still unclear and remains largely hypothetical. We suggest that some communication signals are elevated during cold-stress, thus potentiating aggregation of *L. plantarum* and *B. subtilis* cells (Fig. 4a, e), which can be viewed as a beneficial trait during cross-talks between the probiotic *Bacilli*. The finding envisages *L. plantarum* and *B. subtilis* as prospective candidates for the combined probiotic therapy.

We further addressed the mechanism of consolidated bundle formations, hypothesizing the involvement of cold-shock protein genes (*cspC*, *cspL*, and *cspP*), DnaK, and GroEL, and the molecular chaperones^24^. The molecular chaperones or the heat-shock proteins (HSP) are ubiquitous and conserved in all organisms^24^. In *Lactobacilli*, a single *hsp* gene, *hsp16*, was linked to stress resistance. Interestingly, *L. plantarum* is the only *Lactobacilli* that harbours three different *hsp* genes (*hsp1, hsp2*, and *hsp3*) that assist the bacteria in governing diverse biological functions that coordinate adaptation and survival. The involvement of HSP1 in cryoprotection and improved cryotolerance of *L. plantarum* cells was stated lately^26,27^. Reduced cyroprotection in a *hsp1* knock-out mutant was linked to lower membrane fluidity and reduced biofilm formation^19^. We here show that the mutant exhibits poor ability to form consolidated bundles (Fig. 3a). Overall, the findings support the involvement of HSP1 in cellular cryoprotection.

The acidic-pH (pH 5.5) stressed conic colonies also exhibited better survivability to desiccation stress when compared to colonies generated at the neutral pH (Fig. 1e, f). The result supports our notion that cells exposed to acidic-pH display enhanced survivability. Subsequently, we were further concerned in studying how the bacteria adjust to extremely low pH environments. In general, *L. plantarum* cells are well-adapted to low pH settings^19,26^; hence, the cells undergo rapid physiological reorganization by altering growth or lowering metabolic activities. For instance, Ingham et al. (2008) showed a pH downshift (pH 3) in *L. plantarum* WCFS1 resulted in the formation of elongated (filamentous) cells lacking septation^12^. We report distinctive “V-shaped” geometric cell chains in extremely low pH downshifts (pH 3.5) (Fig. 5a). We also detected a decline in the growth in cells displaying the V-shaped geometry (Fig. 5b) accompanied by a mysterious “bottle effect” when these cells exhibited a strong ability to form biofilms on polystyrene and glass surfaces (Fig. 5c, d & Supplementary Fig. S7). A recent study has shown that the exopolysaccharides (EPS) production could be upregulated in *L. plantarum* cells grown at acidic pH, which supports our observation^28^. Biofilm formation by probiotic species is a propitious tactic to control pathogenic biofilms, which is relatively based on the competitive-exclusion principle^29,30^. It was shown that colonizing LAB may notably reduce the biofilms by *Listeria monocytogenes*, *Salmonella typhimurium*, and *E. coli* O157:H7^31^. We experimented the concept with a pathogenic yeast model, *C. albicans*, and revealed that the “V-shaped” *L. plantarum* cells influenced *C. albicans* biofilm formation (Fig. 5e). *Lactobacilli* were shown to inhibit the early stages of biofilm development in *C. albicans*^32^. Likewise, *Lactobacillus* species are natural competitors of *C. albicans* in the human vaginal environment and are known to dominate the microbiome when the pH is highly acidic (pH 3.5–4.5)^17^. The combination of low pH and lactic acid production by LAB may notably control *C. albicans* infections in vaginal microbiome^33^. We deduce that the dominance displayed by the LAB in low pH condition has possible connections with the V-shaped cell chaining that trigger biofilm growth.

To explore this concept in an in vivo model, we used a *C. elegans*–*C. albicans* co-infection experiment^34–37^. Infections of *C. elegans* with *C. albicans* reduces the life span and longevity of the nematode. On the contrary, feeding *C. elegans* with LABs enhances its life span^37^. In our setup, *C. elegans* fed with V-shaped *L. plantarum* cells exhibited enhanced longevity and defence against *C. albicans* (Fig. 5h)*. L. plantarum* also mitigated *S. aureus*-induced nematode killing (Supplementary Fig. S9), revealing its dominance over bacterial pathogens as well. Overall, the findings shine a light on the dynamic adaptability, dominance, and reign of *L. plantarum* to compete and thrive in hostile pH conditions^34–38^.

## Conclusion

*The probiotic L. plantarum* strains have been vastly known as preserving microbial balance in the human gut through mitigating harmful pathogens directly or by triggering the host immune response reactions. However, they lead an unpredictable lifestyle in their ecological niche and exhibit remarkable potentials to undergo a cellular transformation in response to environmental stresses. The current study provided evidence of how *L. plantarum* expresses numerous cellular phenotypes under acidic-pH conditions. The most intriguing finding appears to be its ability of complex geometrical structuring: cone-shaped colonies, consolidated bundles, and V-shaped chaining. We deduced it as a bio-shielding measure taken by the multicellular bacterial population controlled by a heat-shock response system. Consequently, the unique geometrical arrangement of cells within consolidated bundles could protect the bacterial population from different environmental insults. In addition, the microbial stress recovery concept also applies to the V-shaped structures (Supplementary Fig. S10) because, during stress recovery, a V-shaped cell chain might instantly divide into four cells favouring rapid outgrowth. In terms of growth at acidic pH, *L. plantarum* adopts active cellular conformations and effectually succeeds in prospering. In total, we present the multifaceted traits manifested by probiotic *L. plantarum*, which has significant health implications in the food, pharmaceutical, or medical industries.

## Materials and methods

### Microbial strains and culture media

Specifics of microbial strains used in the study are described in Supplementary Table S1. Microbial strains were cultured and maintained in their respective selective media. For instance, *L. plantarum* was cultured, maintained and experimented in De Man, Rogosa and Sharpe (MRS) (HI media Pvt. Ltd., India) hard agar and/or liquid medium incubated at 37 °C, non-shaking conditions, *B. subtilis* in LB (BD Difco, US) (37 °C, 150 rpm for 5 h)^39^, and *C. albicans* in potato dextrose broth (PDB) (BD Difco, US), (37 °C, 150 rpm, overnight) or PDB supplemented with agar, and/or Roswell Park Memorial Institute medium-1640 (RPMI) (Gibco, US)^40^. The colonies were generated by setting the overnight culture to OD600 = 1 and achieving 10^−7^ dilutions that were subsequently spread on MRS hard agar plates and incubated for either 5, 7, or 15 days at 37 °C. The colony elevation heights and diameters were measured using a standard ruler and a vernier calliper, respectively.

### Growth curve analysis

The *L. plantarum* or *B. subtilis* cells were grown overnight in MRS or LB, respectively, using incubation conditions described above in “Microbial strains and culture media”. The cultures were diluted 1:100 into fresh MRS (pH 3.5 or 5.5) or LB (with or without CSCF) and incubated for 24 h at 37 °C with shaking at 150 rpm (for *B. subtilis*) and non-shaking (for *L. plantarum*). Every 2 h, 1 mL of each sample was collected, and the optical density (OD600) was measured using the Biowave CO8000 cell density meter.

### Crystal violet biofilm quantification assay

The crystal violet staining method was used for the biofilm quantification generated in 48-well microtiter plates (Tarsons Products Pvt. Ltd., India)^40^. Briefly, microbes were inoculated into respective medium and incubated overnight at 37 °C without shaking. Cultures were diluted to OD600 = 0.01 in fresh MRS, re-inoculated into fresh medium, seeded on the microtiter plates, and incubated 37 °C without shaking for 48 h and 72 h. Following incubation, the growth of the cells was measured at optical density (OD) 600 mm using a microtiter plate reader. Then, the cells attached to the surface were stained with 0.1% crystal violet for 20 min, repeatedly washed with sterile distilled water, and suspended in 95% ethanol. Plates were read at 595 nm and OD values were recorded. Graphs were represented as means ± SEMs of four different trials.

### Freeze-thaw challenge of *L. plantarum* conic colonies

An experimental flowchart for *L. plantarum* freeze-thaw challenge assay is depicted in Fig. 2e. Briefly, overnight culture suspensions of *L. plantarum* were diluted to OD600 = 0.01 in fresh MRS broth, spread on MRS hard agar, and incubated for 7 or 15 days at 37 °C. After incubation, the plates were sealed and transferred to −17 °C for 1 h, following which the conic colonies were scrapped off the plate with sterile distilled water (DW). The cells in DW were vortexed vigorously for 5 min and sonicated for 2 min (10 s pulse on/off) at 4 °C with 40% amplitude to break any colony clumps. The samples were visualized under a microscope to make sure there were no clumps in the solution. Then, the cells were incubated at 37 °C and periodically scrutinized under the microscope every 15 min till 3 h for cellular aggregation. The cells were stained with filmtracer™ LIVE/DEAD™ biofilm viability kit (Thermofishers Scientific, US) and visualized under Nikon fluorescent microscope (Nikon Eclipse Ti2, Japan). The quantification of the images (measurement of consolidated bundles intensity) were performed using ImageJ V 1.8.0.

### Cold-shock colony filtrate (CSCF) preparation and assay with unstressed conic colonies of *L. plantarum*

An assay was developed to see if the cold-stressed conic colony (−17 °C for 1 h) extracts could potentiate consolidated bundling in unstressed conic colony cells. Briefly, 1 mL of suspension from harvested cold-stressed (treated) and unstressed (control) conic colonies (~5–6 colonies) were resuspended in DW, vortexed, and sonicated. The extracts were filtered using a 0.2 µm membrane filter to remove the cells. The filtrate was named cold-shock colony filtrates (CSCF). Simultaneously, 1 mL cells from the unstressed colonies were vortexed, sonicated, and pelleted. To these pelleted unstressed cells, 1 mL of CSCF was added, aspirated, and incubated at 37 °C for 3 h. At the same time, colony filtrates (CF) from the unstressed colony and heat-treated (60 °C for 30 min) CSCF were prepared and tested. Following incubation, the aggregation pattern was scrutinized under Nikon fluorescent microscope (Nikon Eclipse Ti2, Japan). As a negative control, one vial of unstressed cells was incubated with 5 M lithium chloride (LiCl_2_) for 30 min before adding 1 mL of CSCF. LiCl_2_ abolishes the S-layer protein and stalls the ability of the cells to aggregate or form bundles.

### pH tolerance assay

*L. plantarum* tolerance to pH was studied by growing the bacteria in the pH gradient ranging from pH 3.5 to 7. Briefly, MRS liquid media and hard agars were prepared, and the pH was adjusted either with 0.1 M NaOH (for alkaline pH) or 1 M HCl (for acidic pH). MRS liquid media with different pH settings were used for assessing the growth profiles on 50-mL Tarson tubes (Tarsons Products Pvt. Ltd., India). The biofilm formations on polystyrene plates as described in “Growth curve analysis”, while the hard agars were used to scrutinize the formation of conic colonies.

### Desiccation stress experiment

The desiccation experiments were performed using the 7-day-old colonies grown on MRS hard agar^21^. Initially, the *L. plantarum* cells were inoculated into liquid MRS and incubated overnight at 37 °C for 24 h. The cultures were then diluted to OD600 = 0.01 in fresh MRS, re-inoculated into a fresh medium, spread on MRS hard agar plates, and incubated for 7 days at 37 °C. The plates were kept open in a desiccation cabinet (MRC, Holon, Israel) at 40% relative humidity and 25 °C for 24 h. Following drying, the whole colony was lifted, suspended in sterile PBS or distilled water, aspirated, sonicated to disrupt colony clumps. Then, the cells were serially diluted and plated on MRS hard agars and incubated at 37 °C for 48 h, following which the colony-forming units (CFU) were counted and recorded.

### Biofilm formation and the matrix expression assays with *Bacillus subtilis*

*B. subtilis* was used as a co-culture probiotic model to examine the biofilm stimulatory effect of cold-shock colony supernatant (CSCF) extracted from *L. plantarum*. For pellicle formation assays, 5 μl of the bacterial suspensions (5 × 10^5^ CFU/mL) were pipetted into 4 mL of LB in 24-well polystyrene plates, following which plates were incubated at 30 °C for 72 h. Images of the pellicles were captured using a smartphone fitted with Leica Vario-Summilux-H1.6–3.4/16–125 ASPH cameras. *B. subtilis* strain YC189 (*PtapA-cfp* harboring strain) was used to assess the biofilm bundles in liquid LB supplemented with or without CSCF (5% v/v and 10% v/v) using CFP filter in a Nikon fluorescent microscope (Nikon Eclipse Ti2, Japan); while the YC121 (*P*_*tapA-lacZ*_ expression) strain was used for assessing the β-galactosidase activity^41^ of the cells collected and resuspended in phosphate-buffered saline (PBS). Typical long bundled chains of cells in the biofilm were disrupted using mild sonication.

### *C. albicans* biofilm inhibition assay with live *L. plantarum*

Effect of live probiotic cells (*L. plantraum* grown at pH 3.5 or 5.5) on *C. albicans* biofilms were tested in *C. albicans* biofilm inducing conditions^42^. Briefly, *C. albicans* biofilms were grown on polystyrene plates for 8 h at 37 °C. After 8 h of incubation, the supernatant was removed, replaced with PBS (controls) or live *L. plantarum* cells in PBS (grown previously in MRS at pH 3.5, or 5.5), and incubated again at 37 °C for 24 h. Biofilm inhibition was then assessed microscopically after staining with SYTO^®^ 9 dye and crystal violet quantification assay.

### *C. elegans* co-infection assays

Wild-type *C. elegans* were maintained on nematode growth medium (NGM) with *E. coli* OP50 as the feed, and synchronization was performed using recently established method^34^. Briefly, *C. elegans* were collected by aspiration and bleached with 2% sodium hypochlorite and 0.5 N sodium hydroxide to get the eggs. Eggs were transferred to 48-well microliter plates and were incubated for 24 h at 22 °C for hatching. The hatched juveniles were transferred to fresh *E. coli* OP50 plates and incubated for 5–7 days to obtain adult nematode. Adults were subsequently used for toxicity, and bacterial or *C. albicans* colonization assays. For *C. albicans* infection of *C. elegans*, the adult nematodes, previously fed on *E. coli* OP50, were transferred to a *C. albicans* lawn on NGM agar plate for 4 h. After 4 h, the nematodes were collected in M9 buffer, pipetted into a 96-well, and survival monitored for 7 days. The treatment groups were adult nematodes previously fed with the probiotic *L. plantarum* lawns prepared from cultures grown at either pH 5.5 or pH 3.5. The live and dead nematodes were counted under bright-field, and DAPi filter and the nematode survival rates were estimated and plotted. The images of *C. elegans* were acquired using a Nikon fluorescent microscope (Nikon Eclipse Ti2, Japan). The sample size were >50 adult nematodes per NGM or 96-well microtitre plate, on average.

### Statistical analysis

All experiments were done in triplicates, and results are expressed as means ± standard deviations with either six or nine individual data points from three biological repeats. The student’s *t*-test was used to determine the significance of differences between treated and non-treated samples. Statistical significance was accepted for *p* values <0.05, and significant changes are indicated using asterisks in figures (*p < 0.05, **p < 0.01, and ***p < 0.001).

### Reporting summary

Further information on research design is available in the Nature Research Reporting Summary linked to this article.

## Supplementary information

Supplementary data

Reporting Summary Checklist

## Data Availability

All relevant data are provided in the manuscript, Supplementary information, or can be obtained from the corresponding author upon reasonable request.

## References

[1] Duar RM (2017). Lifestyles in transition: evolution and natural history of the genus *Lactobacillus*. FEMS Microbiol. Rev..

[2] Martino ME (2016). Nomadic lifestyle of *Lactobacillus plantarum* revealed by comparative genomics of 54 strains isolated from different habitats. Environ. Microbiol..

[3] Jung JH (2019). Multifunctional properties of *Lactobacillus plantarum* strains WiKim83 and WiKim87 as a starter culture for fermented food. Food Sci. Nutr..

[4] Behera SS, Ray RC, Zdolec N (2018). Lactobacillus plantarum with functional properties: an approach to increase safety and shelf-life of fermented foods. Biomed. Res. Int.

[5] Szlufman C, Shemesh M (2021). Role of probiotic *Bacilli* in developing synbiotic food: challenges and opportunities. Front. Microbiol..

[6] Giri SS, Sen SS, Saha S, Sukumaran V, Park SC (2018). Use of a potential probiotic, *Lactobacillus plantarum* L7, for the preparation of a rice-based fermented beverage. Front. Microbiol..

[7] Ramírez MDF, Smid EJ, Abee T, Groot MNN (2015). Characterisation of biofilms formed by *Lactobacillus plantarum* WCFS1 and food spoilage isolates. Int. J. Food Microbiol..

[8] Salas-Jara MJ, Ilabaca A, Vega M, García A (2016). Biofilm forming Lactobacillus: new challenges for the development of probiotics. Microorganisms.

[9] Potočnjak M (2017). Three new *Lactobacillus plantarum* strains in the probiotic toolbox against gut pathogen *Salmonella enterica* serotype *Typhimurium*. Food Technol. Biotechnol..

[10] Foysal MJ, Fotedar R, Siddik MA, Tay A (2020). *Lactobacillus acidophilus* and *L. plantarum* improve health status, modulate gut microbiota and innate immune response of marron (Cherax cainii). Sci. Rep..

[11] Bermúdez-Humarán LG (2019). From probiotics to psychobiotics: live beneficial bacteria which act on the brain-gut axis. Nutrients.

[12] Ingham CJ, Beerthuyzen M, van Hylckama Vlieg J (2008). Population heterogeneity of *Lactobacillus plantarum* WCFS1 microcolonies in response to and recovery from acid stress. Appl. Environ. Microbiol..

[13] Malik S (2013). The highly autoaggregative and adhesive phenotype of the vaginal *Lactobacillus plantarum* strain CMPG5300 is sortase dependent. Appl Environ. Microbiol..

[14] Ferreira CL, Grześkowiak Ł, Collado MC, Salminen S (2011). In vitro evaluation of *Lactobacillus gasseri* strains of infant origin on adhesion and aggregation of specific pathogens. J. Food Prot..

[15] Succi M (2017). Sub-optimal pH preadaptation improves the survival of *Lactobacillus plantarum* strains and the malic acid consumption in wine-like medium. Front. Microbiol..

[16] Hood S, Zoitola E (1988). Effect of low pH on the ability of *Lactobacillus acidophilus* to survive and adhere to human intestinal cells. J. Food Sci..

[17] Zangl I, Pap I-J, Aspöck C, Schüller C (2020). The role of *Lactobacillus* species in the control of *Candida* via biotrophic interactions. Microb. Cell.

[18] Guo L (2020). Effects of lactic acid bacteria isolated from rumen fluid and feces of dairy cows on fermentation quality, microbial community, and in vitro digestibility of alfalfa silage. Front. Microbiol..

[19] Arena MP (2019). The phenotypic analysis of *Lactobacillus plantarum* shsp mutants reveals a potential role for *hsp1* in cryotolerance. Front. Microbiol..

[20] Johnson B, Selle K, O’Flaherty S, Goh YJ, Klaenhammer T (2013). Identification of extracellular surface-layer associated proteins in *Lactobacillus acidophilus* NCFM. Microbiology.

[21] Kimelman H, Shemesh M (2019). Probiotic bifunctionality of *Bacillus subtilis*—rescuing lactic acid bacteria from desiccation and antagonizing pathogenic *Staphylococcus aureus*. Microorganisms.

[22] Warren MR (2019). Spatiotemporal establishment of dense bacterial colonies growing on hard agar. ELife.

[23] Kim W, Racimo F, Schluter J, Levy SB, Foster KR (2014). Importance of positioning for microbial evolution. PNAS.

[24] Liu S (2020). Cold-stress response of probiotic *Lactobacillus plantarum* K25 by iTRAQ proteomic analysis. J. Microbiol. Biotechnol..

[25] Derzelle S (2000). Changes in cspL, cspP, and cspCmRNA Abundance as a function of cold shock and growth phase in *Lactobacillus plantarum*. J. Bacteriol..

[26] Fiocco D, Capozzi V, Goffin P, Hols P, Spano G (2007). Improved adaptation to heat, cold, and solvent tolerance in *Lactobacillus plantarum*. Appl. Microbiol. Biotechnol..

[27] Spano G, Capozzi V, Vernile A, Massa S (2004). Cloning, molecular characterization and expression analysis of two small heat shock genes isolated from wine *Lactobacillus plantarum*. J. Appl. Microbiol..

[28] Nguyen P-T, Nguyen T-T, Hoang Q-K, Nguyen H-T (2021). Response of *Lactobacillus plantarum* VAL6 to challenges of pH and sodium chloride stresses. Sci. Rep..

[29] Falagas M, Makris G (2009). Probiotic bacteria and biosurfactants for nosocomial infection control: a hypothesis. J. Hosp. Infect..

[30] Hibbing ME, Fuqua C, Parsek MR, Peterson SB (2010). Bacterial competition: surviving and thriving in the microbial jungle. Nat. Rev. Microbiol..

[31] Gómez NC, Ramiro JM, Quecan BX, de Melo Franco BD (2016). Use of potential probiotic lactic acid bacteria (LAB) biofilms for the control of *Listeria monocytogenes, Salmonella typhimurium*, and *Escherichia coli* O157: H7 biofilms formation. Front. Microbiol..

[32] Matsubara VH, Wang Y, Bandara H, Mayer MPA, Samaranayake LP (2016). Probiotic *lactobacilli* inhibit early stages of *Candida albicans* biofilm development by reducing their growth, cell adhesion, and filamentation. Appl. Microbiol. Biotechnol..

[33] K”hler GA, Assefa S, Reid G (2012). Probiotic interference of Lactobacillus rhamnosus GR-1 and Lactobacillus reuteri RC-14 with the opportunistic fungal pathogen Candida albicans. Infect. Dis. Obstetr. Gynecol.

[34] Rajasekharan SK, Raorane CJ, Lee J (2018). LED based real-time survival bioassays for nematode research. Sci. Rep..

[35] Hunt PR (2017). The *C. elegans* model in toxicity testing. J. Appl. Toxicol..

[36] Scanlan LD (2018). Counting *Caenorhabditis elegans:* protocol optimization and applications for population growth and toxicity studies in liquid medium. Sci. Rep..

[37] Poupet C, Chassard C, Nivoliez A, Bornes S (2020). Caenorhabditis elegans, a host to investigate the probiotic properties of beneficial microorganisms. Front. Nutr.

[38] Breger J (2007). Antifungal chemical compounds identified using a *C. elegans* pathogenicity assay. PLoS Pathog..

[39] Ben-Ishay N (2017). Enrichment of milk with magnesium provides healthier and safer dairy products. NPJ Biofilms Microbiomes.

[40] Rajasekharan SK, Lee J-H, Lee J (2019). Aripiprazole repurposed as an inhibitor of biofilm formation and sterol biosynthesis in multidrug-resistant *Candida albicans*. Int. J. Antimicrob. Agents.

[41] Shemesh M, Chai Y (2013). A combination of glycerol and manganese promotes biofilm formation in *Bacillus subtilis* via histidine kinase KinD signaling. J. Bacteriol..

[42] James K, MacDonald K, Chanyi R, Cadieux P, Burton J (2016). Inhibition of *Candida albicans* biofilm formation and modulation of gene expression by probiotic cells and supernatant. J. Med. Microbiol..

