# Supplementary material for: Spatiotemporal bio-shielding of bacteria through consolidated geometrical structuring
Source: NPJ Biofilms Microbiomes. 2022 May 9;8:37. doi: 10.1038/s41522-022-00302-2 (PMC9085766; doi:10.1038/s41522-022-00302-2)
Supplement: Supplementary file 1 — Supplementary data [file 41522_2022_302_MOESM1_ESM.doc]

**Supplementary information**

**Spatiotemporal bio-shielding of bacteria through consolidated** **geometrical structuring**

Satish Kumar Rajasekharan, and Moshe Shemesh*

^1^ Department of Food Science, Institute of Postharvest Technology and Food Sciences, Agricultural Research Organization (ARO), The Volcani Institute, Rishon LeZion 7528809, Israel.

***Corresponding author:** moshesh@agri.gov.il

**Supplementary Tables**

**Supplementary Table S1. Microbial strains used in this study.**

| **Strain** | **Specifications** | | **Reference** |
| --- | --- | --- | --- |
| *B. subtilis* NCIB3610 | Wild-type strain | (Branda, et al., 2001) | |
| *B. subtilis* YC189 | P*_tapA-cfp_* in 3610 | (Chai, et al., 2011) | |
| *B. subtilis* YC121 | P*_tapA-_LacZ* in 3610 | (Chen et al., 2015) | |
| *L. planarum* 3297 | Isolate from healthy cow | This study | |
| *C. albicans* SC5314 | Clinical specimen - human | (Feldman et al., 2017) | |
| *E. coli* OP50 | Uracil auxotroph,  Feed for *C. elegans* | (Sanadaya et al., 2018) | |
|  |  |  | |
|  |  |  | |
|  |  |  | |
|  |  |  | |
|  |  |  | |
|  |  |  | |

**Supplementary Figures**

**
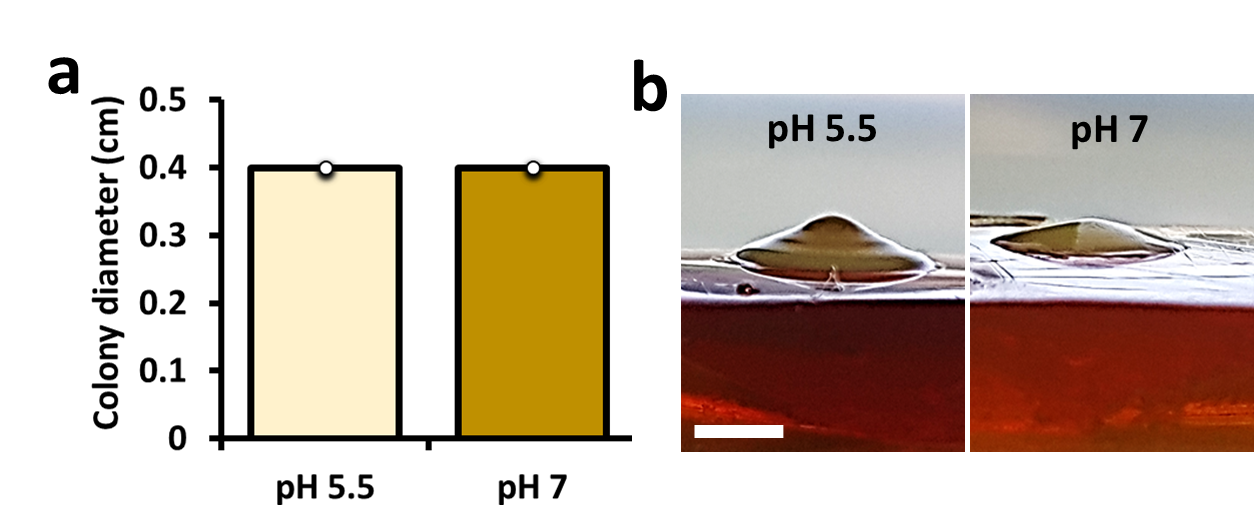
**

**Supplementary Fig. S1. Assessment of *L. planatrum* colony parameters.**

1. Estimation of *L. plantarum* colony diameters grown on MRS hard agar with different pH conditions. The graph shows the means ± SEMs of three measurements. **P >* 0.05 vs. the non-treated controls.
2. Representative image showing height difference between colonies grown on pH 7 and pH 5.5 MRS hard agars. Scale bar: 0.2 cm.

**
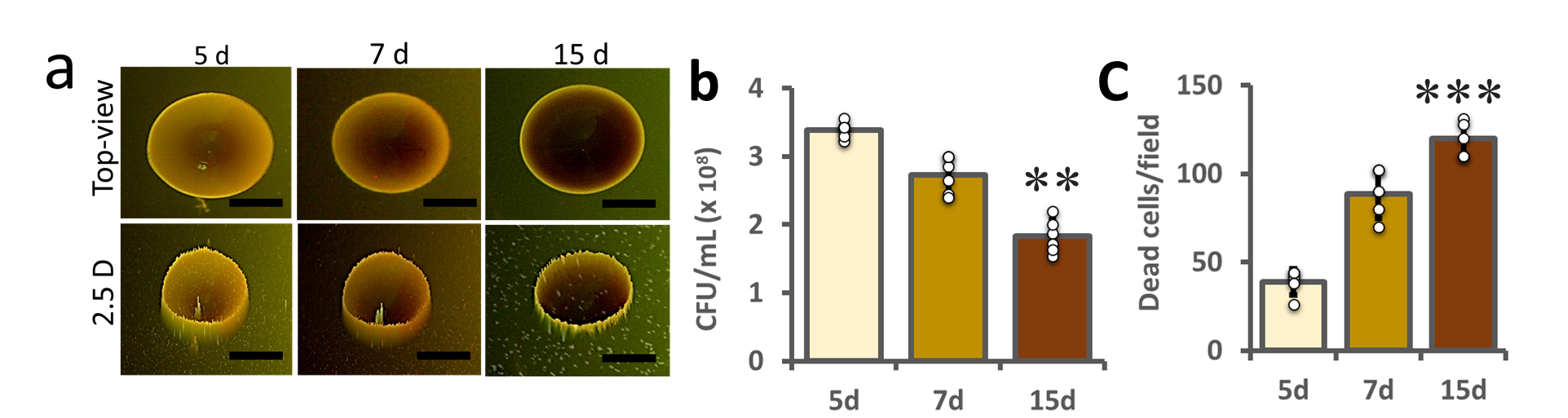
**

**Supplementary Fig. S2. Effect of colony aging on brown coloration within the colony.**

1. Microscopic image of aging colonies of *L. plantarum* 3297 that show increased accumulation of brown matrix during prolonged incubation. Scale bar: 0.2 cm.
2. Colony forming units (CFUs) of *L. plantarum* grown at pH 5.5 after 5, 7 and 15 days of incubation. Whole colony (with similar diameter) was lifted, diluted in PBS and plated again on MRS agar. ** *P*<0.01 vs. 5d colonies.
3. Quantitative profile of dead cells (stained with propidium iodide (PI)) in the aged conic colony of *L. plantarum*. The graph shows the means ± SEMs of three measurements. *** *P*<0.001 vs. 5d colonies.


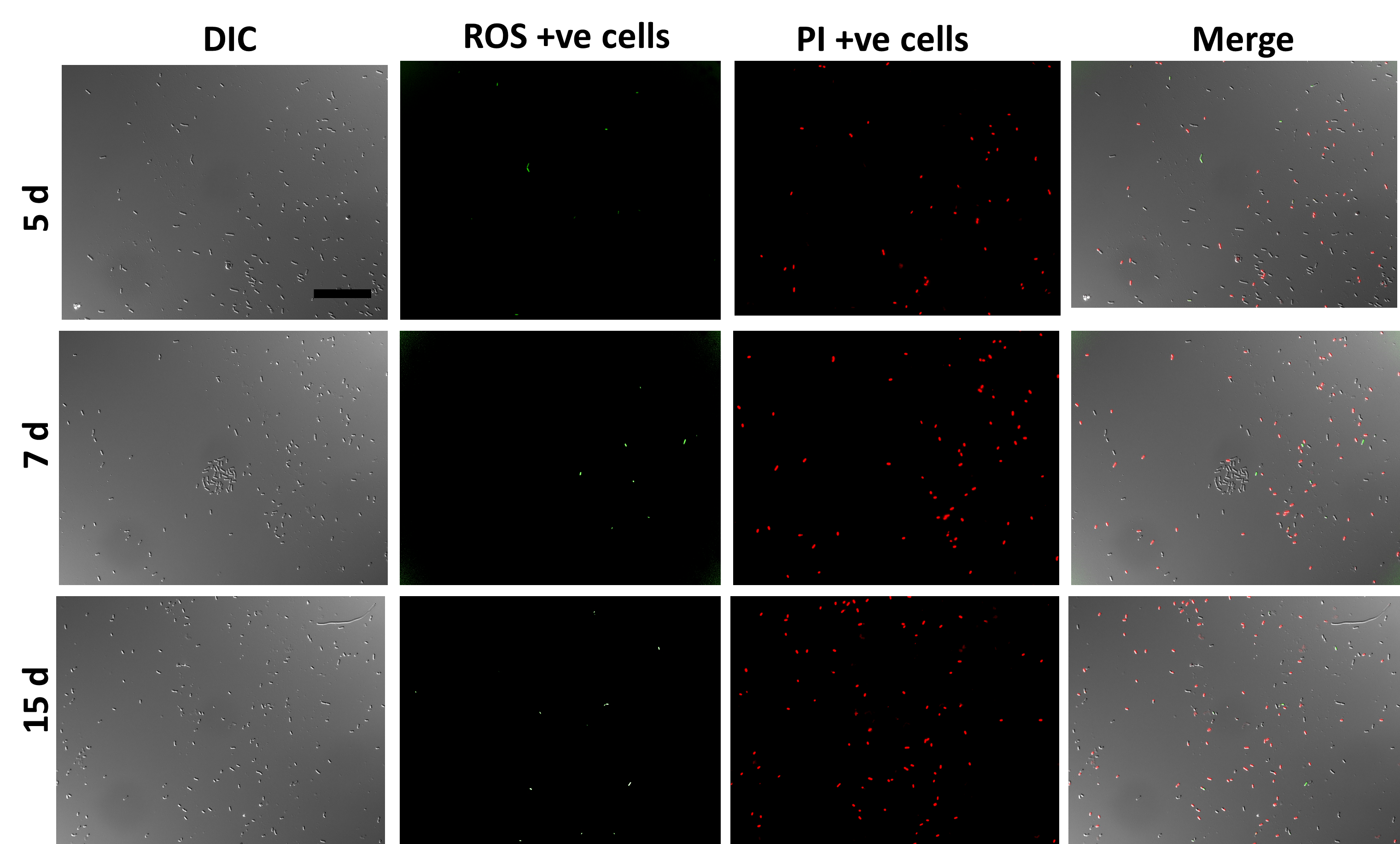
**Supplementary Fig. S3. Effect of colony aging on brown coloration within the colony.** Microscopic images of reactive oxygen species (ROS) positive (green), PI positive (red) and live cells (unstained in merge) in an ageing colony. Scale bar: 20 µm.


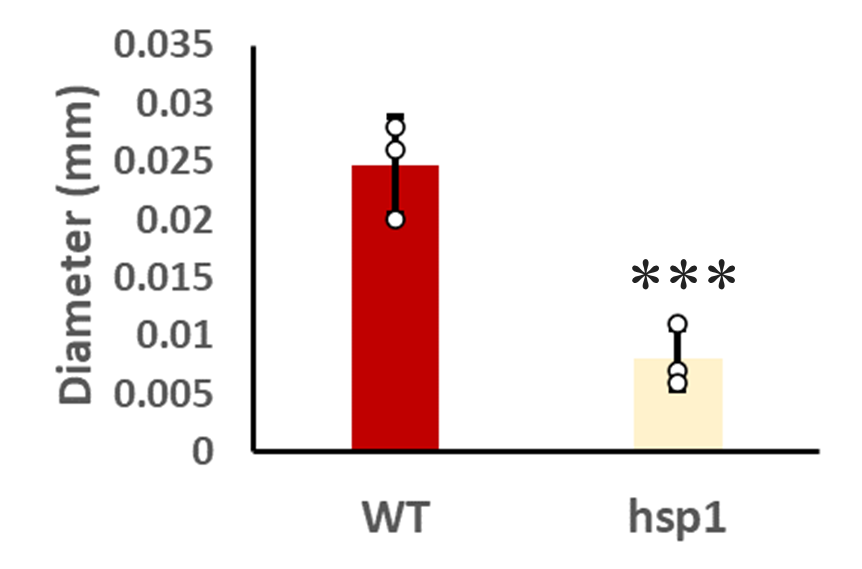


**Supplementary Fig. S4. Mean diameters of the circular bundles formed by WT and *hsp1* mutant.** The graph shows the means ± SEMs of three measurements. *** *P*<0.001 vs. WT control.

**
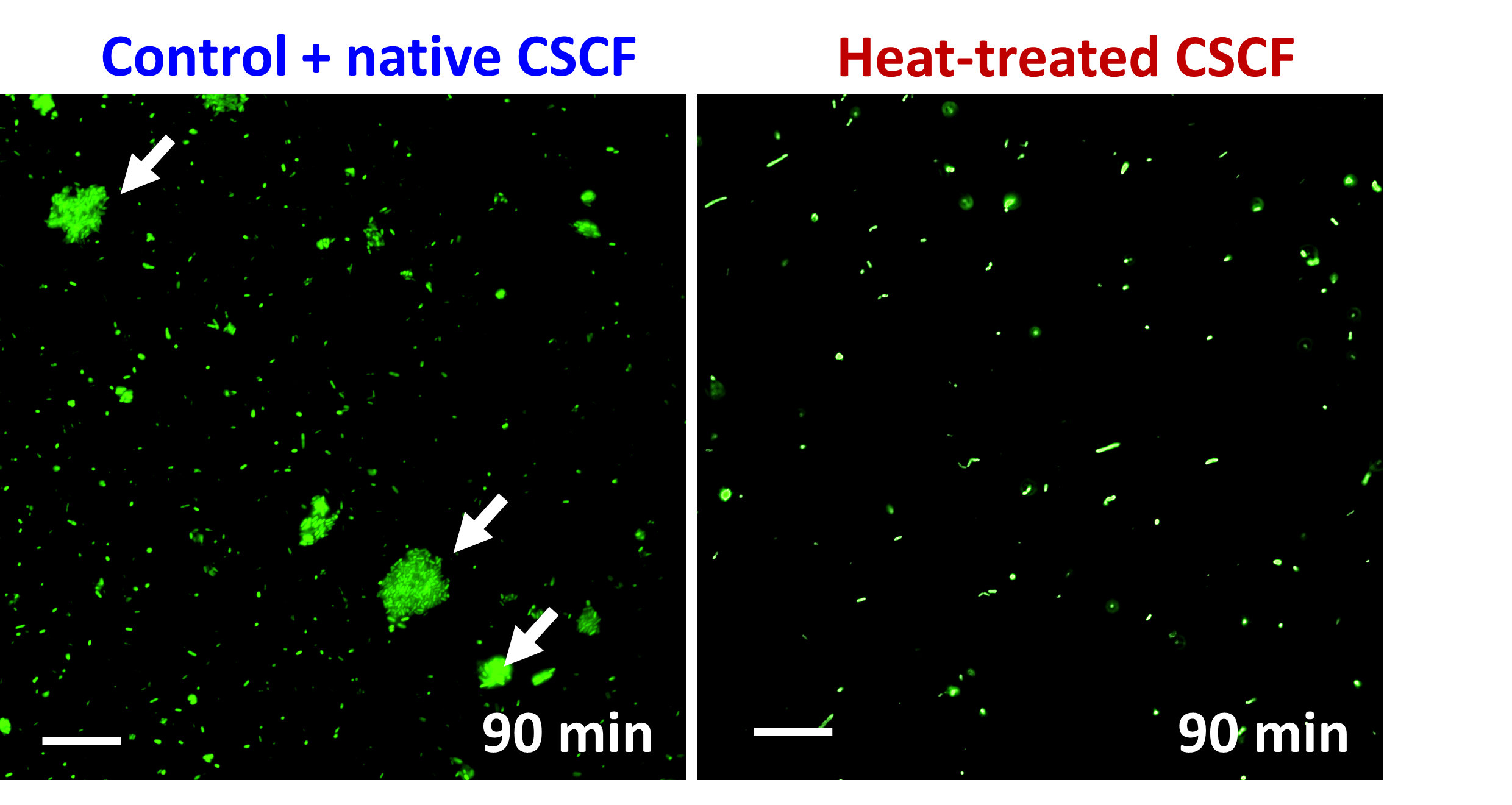
Supplementary Fig. S5. Effect of heat treated (60°C for 30 min) colony filtrates (CF) on circular bundle formation (b). Scale bar: 20 µm. CSCF stands for ‘cold-shock colony filtrate’.**

**
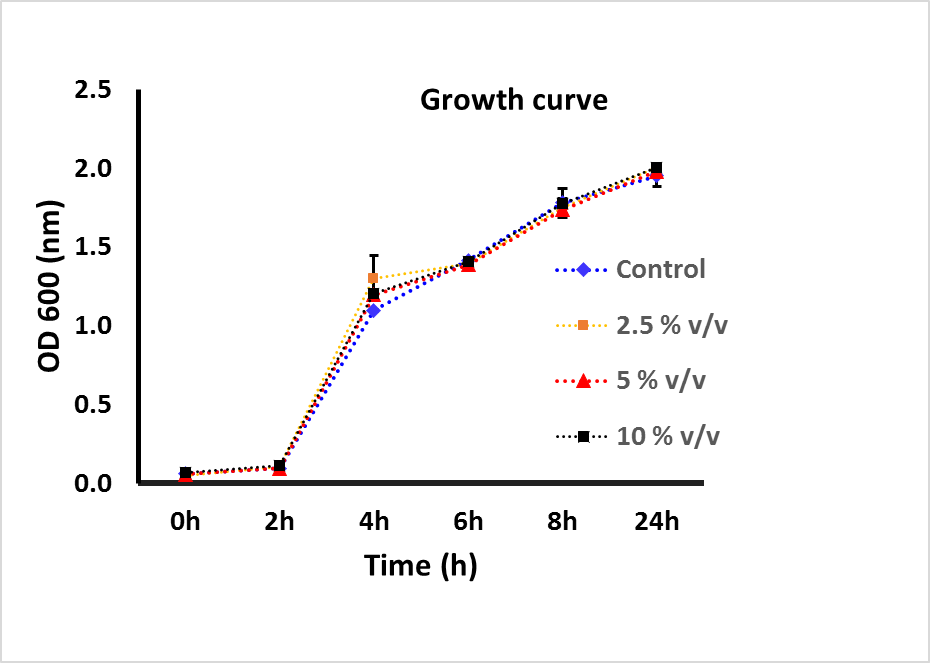
**

**Supplementary Fig. S6. Growth curve analysis of *B. subtilis* in the presence and absence of CSCF.** **v/v denotes volume per volume.**


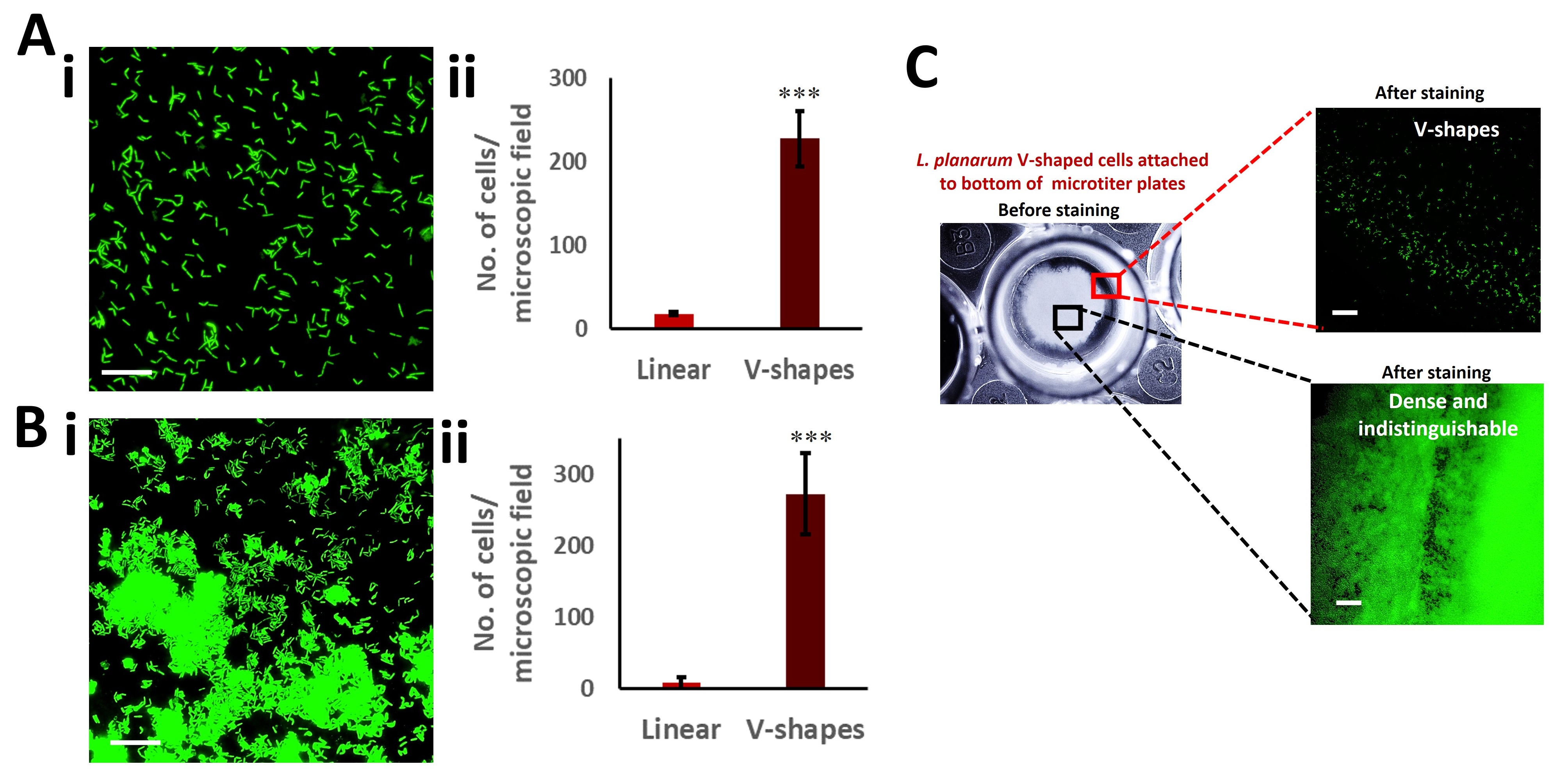


**Supplementary Fig. S7: Effect of low pH on biofilm formation by *L. planatrum.***

1. Qualitative (i) and quantitative data (ii) showing the ratio of V-shaped and linear *L. planatrum* cells grown in liquid broth (pH 3.5, for 24 h at 37°) and transferred to slides for microscopy. Scale bar: 20 µm. Magnification: 1000X (100X objective x 10X ocular). The graph shows the means ± SEMs of three measurements. *** *P*<0.001 vs. the linear cells.
2. Qualitative (i) and quantitative data (ii) showing the ratio of V-shaped and linear *L. plantarum* cells grown directly as biofilms on glass coverslips. For this experiment, a glass coverslip was dropped on the surface of a 12-well microtiter plate and grown with the MRS media (pH 3.5). After incubation for 48 h, the slides were carefully taken out, washed, stained with SYTO9 and imaged with florescent microscope. Scale bar: 20 µm. Magnification: 1000X (100X objective x 10X ocular). The graph shows the means ± SEMs of three measurements. *** *P*<0.001 vs. the linear cells.
3. Microscopic image of cells grown directly on 24-well microtiter plates in liquid broth (pH 3.5, incubated for 48 h at 37°). The biofilms on the polystyrene surface (before fluorescent staining, but after washing procedure) of the microtiter plate is shown. Black inset box and dashed lines show the biofilms at the centre following staining. The area is dense and it is not possible to distinguish the V-shapes. The red box and dashed lines show the biofilms on the periphery where there were less cells and here the V-shapes can be clearly distinguished. Scale bar: 100 µm. Magnification: 100X (10X objective x 10X ocular).

***
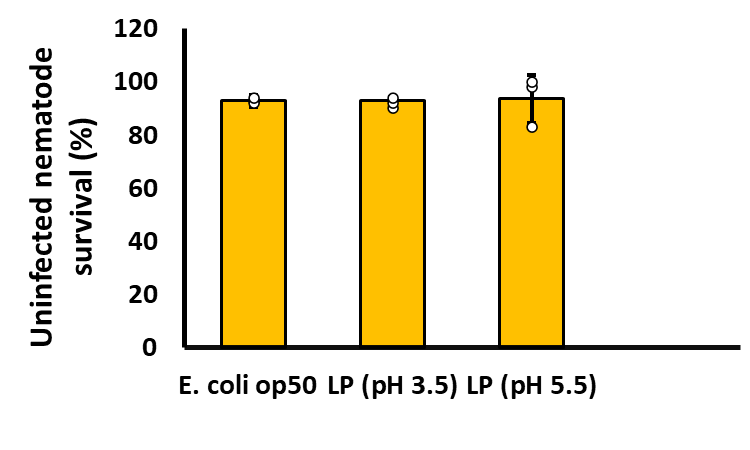
***

**Supplementary Fig. S8.** Survival rates of uninfected nematodes fed with *E. coli* OP50, and *L. plantarum* cells grown at pH 3.5 or 5.5. The graph shows the means ± SEMs of three measurements. **P >* 0.05 vs. the control.

***
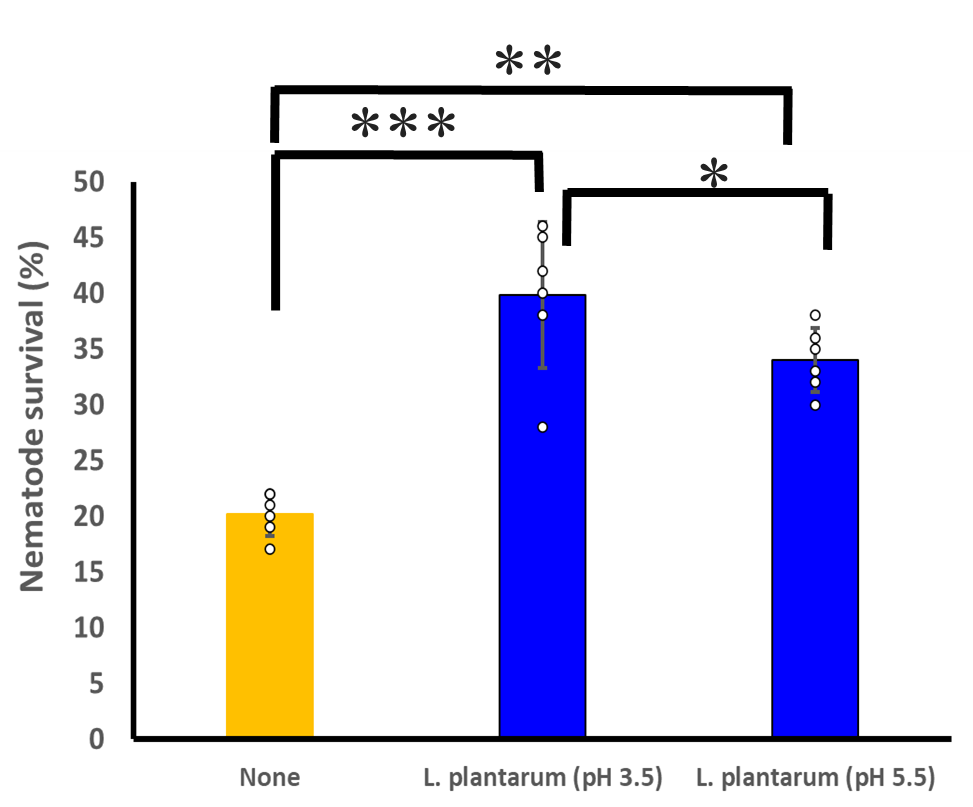
***

**Supplementary Fig. S9. Effect on live probiotics on survival of *C. elegans* infected with *S. aureus.*** The graph shows the means ± SEMs of three measurements. * *P*<0.05 significance for *L. plantarum* (pH 3.5) vs *L. plantarum* (pH 5.5), and ** *P*<0.01, *** *P*<0.01 vs. the *E. coli* control.


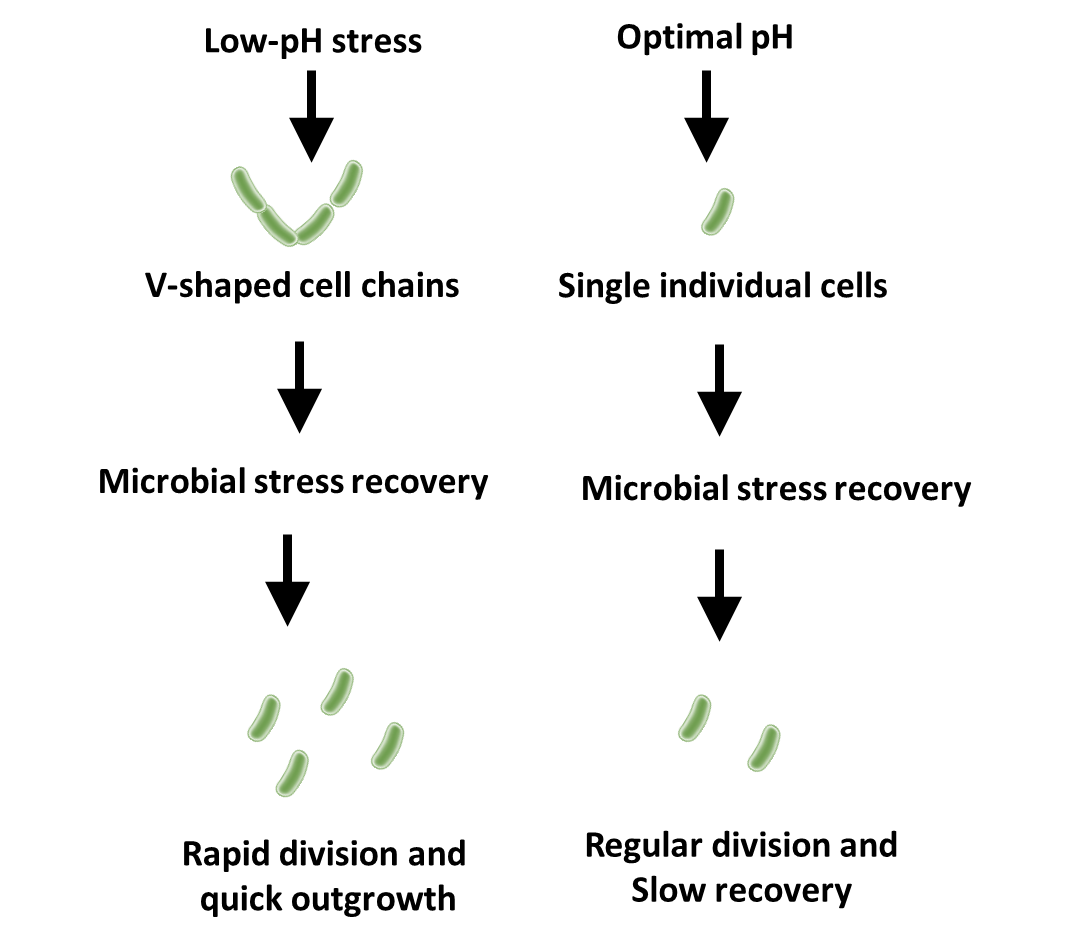


**Supplementary Fig. 10. A model depicting microbial stress recovery in V-shaped and regular cells.**

**Supplementary references**

Branda, S.S., González-Pastor, J.E., Ben-Yehuda, S., Losick, R., and Kolter, R. (2001) Fruiting body formation by *Bacillus subtilis*, *Proceedings of the National Academy of Sciences* **98**: 11621-11626.

Chai, Y., Norman, T., Kolter, R., and Losick, R. (2011) Evidence that metabolism and chromosome copy number control mutually exclusive cell fates in *Bacillus subtilis*, *The EMBO Journal* **30**: 1402-1413.

Chen Y, Gozzi K, Yan F, Chai Y. Acetic Acid Acts as a Volatile Signal To Stimulate Bacterial Biofilm Formation. mBio. 2015;6(3):e00392

Sanadhya, P., Bucki, P., Liarzi, O., Ezra, D., Gamliel, A., & Braun Miyara, S. (2018). *Caenorhabditis elegans* susceptibility to Daldinia cf. concentrica bioactive volatiles is coupled with expression activation of the stress-response transcription factor daf-16, a part of distinct nematicidal action. PloS one, 13(5), e0196870.

Feldman M, Shenderovich J, Lavy E, Friedman M, Steinberg D. A Sustained-Release Membrane of Thiazolidinedione-8: Effect on Formation of a Candida/Bacteria Mixed Biofilm on Hydroxyapatite in a Continuous Flow Model. Biomed Res Int. 2017;2017:3510124. doi:10.1155/2017/3510124

Kimelman H., Shemesh M. (2019). Probiotic Bifunctionality of *Bacillus subtilis* - Rescuing Lactic Acid Bacteria from Desiccation and Antagonizing Pathogenic *Staphylococcus aureus*. *Microorganisms* 7 (10):407.
